# *Bryophyllum pinnatum* and Improvement of Nocturia and Sleep Quality in Women: A Multicentre, Nonrandomised Prospective Trial

**DOI:** 10.1155/2023/2115335

**Published:** 2023-02-07

**Authors:** Nurlana Mirzayeva, Susanne Forst, Daniel Passweg, Verena Geissbühler, Ana Paula Simões-Wüst, Cornelia Betschart

**Affiliations:** ^1^Department of Gynaecology, University Hospital and University Zurich, Zurich 8091, Switzerland; ^2^Department of Obstetrics and Gynaecology, Cantonal Hospital Aarau, Aarau 5001, Switzerland; ^3^Department of Obstetrics and Gynaecology, Cantonal Hospital Winterthur, Winterthur 8400, Switzerland; ^4^Department of Obstetrics and Gynaecology, Triemli Hospital, Zurich 8063, Switzerland; ^5^Department of Gynaecology, St. Clarapsital, University Basel, Basel 4058, Switzerland; ^6^Department of Obstetrics, University Hospital Zurich, University of Zurich, Zurich 8091, Switzerland; ^7^Clinic Arlesheim, Research Department, Arlesheim 4144, Switzerland

## Abstract

Nocturia is a pathologic condition that significantly affects the quality of sleep. The aetiology of nocturia is multifactorial, and the evidence available on its management remains limited. Besides behavioural measures, validated pharmaceutical treatment options exist but are, however, associated with marked side effects. Prospective clinical studies with tablets prepared from the leaf press juice of the plant *Bryophyllum pinnatum* revealed a tendency towards reduction of micturition in patients with overactive bladder (OAB) and several improvements in sleep quality. These observations are in part supported by in vitro and in vivo data. In the present study, we investigated the effectiveness of Bryophyllum 50% chewable tablets in the treatment of nocturia and associated sleep disorders. Altogether, 49 women with idiopathic OAB and nocturia of ≥2 voids/night were treated with Bryophyllum 50% tablets for 3 weeks (350 mg chewable tablets, dosage 0-0-2-2 oral tablets; WELEDA AG, Arlesheim, Switzerland). Nocturia, voiding volumes at night (ml), quality of life, sleep quality, and daily sleepiness were assessed before and after treatment with a 3-day micturition diary, the International Consultation on Incontinence evaluating overactive bladder and related impact on quality of life (QoL) [ICIQ-OAB], the Pittsburgh Sleep Quality Index (PSQI), and the Epworth Sleepiness Scale (ESS), respectively. The age of the study population was 68.5 ± 11.6 y. After treatment, nocturia diminished from 3.2 ± 1.4 to 2.3 ± 1.3 (*P*  <  0.001) and the PSQI score decreased from 7.7 ± 3.7 to 6.6 ± 3.4 (*P*=0.004). Urgency, the ICIQ score, and the ESS lowered significantly, and the micturition volume showed a tendency to increase. No serious adverse drug reactions were reported, and compliance was good. The results show a beneficial effect on the nocturnal voids and sleep quality of women with OAB. Bryophyllum 50% tablets can be regarded as a well-tolerated alternative in the treatment of nocturia and broaden the repertoire of standard management.

## 1. Introduction

Night-time micturition, called nocturia, is a common problem that unfavourably influences both sleep and life quality [1]. The International Continence Society defines nocturia as urinating once or several times during the night [2]. A period of sleep, or attempted sleep, must precede and follow the urinary episode to count as a nocturnal void [3]. Nocturia is one of the cardinal symptoms of overactive bladder (OAB) [4]. Contrary to cases of diurnal urgency, persons suffering from nocturia intend to continue sleeping after going to the toilet but do not always succeed, an outcome that often depends on their age.

The aetiology of nocturia is multifactorial, with causes such as hypertension, heart disease, diabetes mellitus, lower limb oedema, and lung disease, which are commonly associated with polyuria, as well as neurological diseases and urinary incontinence [5]. Nocturia significantly affects quality of sleep and leads long-term to increased morbidity and mortality in women with idiopathic overactive bladder [6]. There is also a substantial economic impact on health care, with annual direct costs of healthcare resources of approximately €2.32 billion in Germany, €0.54 billion in Sweden, and €1.77 billion in the UK [7]. Besides behavioural measures, validated treatment options include oral desmopressin, diuretics, anticholinergics, botulinum toxin, and sedatives [8]. Since these pharmaceutical options are associated with side effects such as hyponatremia, dizziness, dry mouth, and fatigue, and given the chronic nature of nocturia, compliance with these treatments is low, and their use in elderly persons is often not advisable [9–11].

*Bryophyllum pinnatum* (Lam.) Oken is a plant of the family Crassulaceae. It is known by various common names, such as life plant, air plant, love plant, cathedral bells, and Goethe plant. The plant occurs naturally in Madagascar and grows well in tropical Africa, America, India, Asia, and Australia. In folk medicine in these countries, it is widely used in the treatment of kidney and urinary diseases, ulcers, diarrhoea, cough, asthma (as prophylaxis), jaundice, wounds, bruises, and as an antipyretic [12, 13]. These indications are in line with some of the known effects of the leaf extracts from *B. pinnatum,* which have been shown to possess diuretic, gastroprotective, anti-inflammatory, antiseptic, analgesic, immunomodulating, anticancer, and wound-healing properties [14–18]. In Europe, the use of *Bryophyllum* spp. was introduced into anthroposophic medicine at the beginning of the 20^th^ century. In this type of wholistic medicine, it is still often used for sleep and anxiety disorders [19] and, since the 1970s, for the treatment of preterm labour and other hyperactivity diseases [13]. In Switzerland, its use during pregnancy to prevent preterm uterus contractions is well established [20, 21], and uterus-relaxant properties have been proven at both the tissue [22, 23] and cell level [24, 25]. Some clinical studies have revealed its potential in the treatment of sleep disorders, be they during pregnancy [26] or associated with other diagnoses [27, 28]. Its use in these indications corresponds well to the sedative, central nervous system depressant, and anxiolytic properties that have been shown in animal models, namely rats, mice [29], and zebra fish [30].

More recently, *B. pinnatum* preparations have begun to be considered in the treatment of OAB. A prospective clinical study with *B. pinnatum* 50% chewable tablets prepared from leaf press juice revealed a tendency towards the reduction of micturition in patients with OAB [31] and very good tolerability among the group of patients studied. The plausibility of these clinical data regarding efficacy was supported by in vitro data that revealed relaxant effects of several B. pinnatum compounds on detrusor muscle [32, 33]. In view of the clinical and in vitro observations, the known sedative effects, and the favourable side-effect profile, we hypothesized that *B. pinnatum* could have potential in the long-term treatment of nocturia and associated sleep disorders. In the present prospective study with a pre/post design, we therefore investigated the effectiveness of *B. pinnatum* 50% chewable tablets on subjective and objective outcome parameters in the treatment of those disorders.

## 2. Material and Methods

### 2.1. Study Patients

Forty-nine women, aged >18 years, with idiopathic OAB were included in this study. Additional inclusion criteria were OAB combined with urge, >8 mictions per day, and nocturia ≥2 voids/night, hence disrupted sleep. Patients who did not fulfil all the inclusion criteria were excluded. Further exclusion criteria were neurogenic hyperactive bladder, hypersensitivity to the study drug or its active compound or to drugs similar to the study drug, lactose intolerance, and the intake of bladder active drugs (anticholinergics, diuretics, bladder relaxing drugs including other phytomedicines) less than 4 weeks prior to study begin. Further exclusion criteria were acute UTI, pregnancy or lactation, drug or alcohol abuse, malcompliance, and participation in another study (current or during the last 4 weeks prior to inclusion in this study). Local systemic HRT was acceptable when started more than 4 weeks before study enrolment. A prior incontinence operation had to date back more than 6 months. Study information and informed consent were available only in German; hence, only German-speaking women were enrolled.

### 2.2. Study Medication

All participants were treated with Bryophyllum 50% (chewable tablets of 350 mg, each corresponding to 170 mg of fresh leaf press juice). No randomization into treatment and control groups took place. Bryophyllum was administered to all patients for 3 weeks: 2 tablets with supper and 2 tablets before going to bed (a total of 4 tablets/day).

The chewable tablets were manufactured by WELEDA AG, Arlesheim, Switzerland, with *B. pinnatum* leaves provided by Weleda, Brazil, and are registered at the Swiss Agency for Therapeutic Products (Swissmedic; without indication). A voucher specimen (Nr. ZSS 29717) has been deposited at the Zurich Succulent Plant Collection, Switzerland. Previous analytical work on the press juice of fresh or frozen *B. pinnatum* leaves, which are routinely used to manufacture Bryophyllum 50% chewable tablets, revealed the presence of flavonoids as major constituents, along with cinnamic acid derivatives and bufadienolides [22].

### 2.3. Endpoints (Outcomes)

#### 2.3.1. Primary Endpoints

- reduction of nocturia frequency (3-day voiding diary)
- improvement of sleep quality (Pittsburgh Sleep Quality Index (PSQI))

#### 2.3.2. Secondary Endpoints

- improvement of the bladder specific quality of life (International Consultation on Incontinence Questionnaire Overactive Bladder (ICIQ-OAB))
- reduction of sleepiness score (Epworth Sleepiness Scale (ESS))
- assessment of AE/SAE; safety

### 2.4. Measurements

Patients filled out a 3-day voiding diary that includes fluid intake, frequency of voiding, urine volume in 24 hours (ml), urine volume at night (ml), urge intensity, urge episodes (+-+++), incontinence episodes (y/n), nocturia index (Ni), nocturnal polyuria index, and nocturnal bladder capacity index (NBCi).

The nocturia index (Ni) is calculated as nocturnal urine volume (NUV) divided by the maximal voiding volume for 24 hours; the nocturia index is defined as positive if it is greater than 1 Nocturnal polyuria is defined as NUV >33% of the total 24-h urine volume at the age of 65 years and higher. The NBCi corresponds to the actual number of voids minus the predicted number of voids. The predicted number of voids is obtained by subtracting 1 from the nocturia index (Ni) [34].

Four detailed questionnaires were filled out by the participants for all 7 days before the treatment with Bryophyllum 50% chewable tablets and during the last days of the 3-week-long treatment. For measuring the primary outcomes, nocturia and sleep quality, the averages of voiding at night from the 3-day voiding diary and the Pittsburgh Sleep Quality Index (PSQI, Questionnaire 1) were used. The PSQI is a self-report questionnaire that assesses sleep quality. It consists of 19 individual items that create 7 components that produce one global score (0–21) and takes 5–10 minutes to complete. These components (each one rated 0–3) are subjective sleep quality (component 1), sleep latency (component 2), sleep duration (component 3), habitual sleep efficiency (component 4), sleep disturbance (component 5), use of sleeping medication (component 6), and daytime dysfunction (component 7). The higher the score, the higher the symptom burden.

For the assessment of the secondary outcomes, the International Consultation on Incontinence Questionnaire-OverActive Bladder (ICIQ-OAB, Questionnaire 2) was used. This is a questionnaire for evaluating overactive bladder, its related impact on quality of life (QoL), and the outcomes of treatments in men and women in research and clinical practice. Higher values in the overall 0–16 score indicate increased symptom severity.

To assess daytime sleepiness as a sequela of night-time sleep disturbance, the Epworth Sleepiness Scale (ESS) (Questionnaire 3), which consists of 8 questions, was used. Adding the scores for each of the 8 questions yields a total score ranging from 0–24. An ESS score >10 suggests excessive daytime sleepiness (EDS); an ESS score ≥16 suggests a high level of EDS and is usually associated with marked sleep disorders, including narcolepsy.

Finally, the patients filled out a customized questionnaire (Questionnaire 4) on a daily basis to record discomforts and adverse events that they thought could be associated with the treatment and to characterize their tablet intake (forgotten tablets had to be registered); this allowed for the assessment of safety parameters and patient compliance.

### 2.5. Statistics

By analogy to our previous observational studies in oncology and obstetrics on sleep quality and *B. pinnatum,* the study was designed to include a maximum of 50 patients [26, 27]. At analysis, one patient had to be excluded due to a double entry in the database. Regarding nocturia, no observational study with *B. pinnatum* is available on which a power analysis could have been performed. Data were analysed using the IBM SPSS Statistics software for Windows, version 24. Overall, *p* values were calculated by the student's *t*-test for paired samples. A *p* value <0.05 was considered significant. Data are expressed as mean ± standard deviation (SD). The number of valid pairs is indicated if less than 49; missing values were not replaced.

## 3. Results

### 3.1. Study Population and Baseline Characteristics

Altogether, 49 women were included from the 63 patients screened. All participants were treated with Bryophyllum 50% chewable tablets. The average age of the study population was 68.5 ± 11.6 years. The number of tablets taken by the patients was on average 79.1 ± 9.8 (maximum 84 tablets). Detailed demographic, safety, and compliance data of the female study collective is shown in Table 1.

### 3.2. Efficacy

A significant reduction of nocturia (−0.9) was recorded in the 3-day voiding diary, and improvements in the specific questionnaire on sleep quality (PSQI), overall and the subdomains of sleep quality, sleep duration, and daytime dysfunction could be demonstrated (Table 2).

For the secondary outcomes, a significant reduction in nocturnal and 24-hour micturition (ICIQ-OAB, questions 1 and 2) was observed upon treatment with Bryophyllum 50% chewable tablets (Table 3).

The same results on nocturnal voiding were reflected in the objective 3-day voiding diary (Table 4). The ICIQ-OAB global score was also significantly reduced, which demonstrates an effect on the whole overactive bladder syndrome and the bladder-related impact on quality of life (QoL).

The specific questionnaire on daytime sleepiness (ESS), which rated the propensity to doze or fall asleep during common daily activities, revealed significant improvements in the level of daytime sleepiness during treatment (Table 3). Urge symptoms and incontinence improved, but not significantly.

Additionally, we examined the micturition characteristics before and after treatment to better describe the study population. Along with the reduction in nocturia, fluid intake and micturition per 24 hours also decreased. The nocturia index (Ni), the nocturnal polyuria index, and the nocturnal bladder capacity index (NBCi) showed pathologic results before treatment and improved significantly after treatment, with *p* values of <0.001, 0.034, and 0.036, respectively (Table 4).

### 3.3. Safety

No serious adverse event (SAE) was detected. Perceived discomforts (*n* = 8) were bladder-related in three cases (two urinary tract infections and one de novo incontinence), and in six cases, single findings included hypotonia, leg spasms, stomach pain, and whole-body itching (each symptom reported by a different patient). Two of the eight events were classified as possible suspected adverse drug reactions (SADR), namely stomach pain and whole-body itching, which both might have been evoked by drug reactions.

## 4. Discussion

This prospective pre/post study of Bryophyllum 50% chewable tablets for the treatment of nocturia showed a beneficial effect on the number of nocturnal voids as well as on the life and sleep quality of women with OAB. After treatment, nocturia was significantly reduced (from 3.2 ± 1.4 to 2.3 ± 1.3 voids per night; *p* < 0.001) and sleep quality significantly improved (PSQI score decreased from 7.7 ± 3.7 to 6.6 ± 3.4; *p* = 0.004). Urgency, the ICIQ score, and the ESS lowered significantly, and the micturition volume showed a tendency to increase. No serious adverse drug reactions were reported, and only a few discomforts possibly associated with the treatment were reported by patients; patient compliance with taking the study drug and recording measures was high. Taken together, the results show a beneficial effect on the nocturnal voids and sleep quality of women with OAB.

Treatment of nocturia overall is challenging: a meta-analysis of anticholinergics that are widely used for the treatment of OAB-associated nocturia showed improvement by reducing nocturia episodes by 0.5 episodes per night [34]. A higher efficacy was demonstrated by Weiss et al. in a 12-week prospective, double-blind study, where nocturnal micturition per 24 hours decreased by −1.02 after the intake of fesoterodine (verum) and by −0.85 in the placebo group [35]. However, in a recent narrative review, placebo reduced nocturia by 0.2–0.4 voids per night [36]. The slight reduction in fluid intake recorded in the 3-day diary might be interpreted as a learning effect to prevent polakisuria; however, the reduction was not significant in our collective. With an average fluid intake of less than 2 liters before and after *B. pinnatum*, our study population collectively represents a conventional fluid intake population for the majority of females. In the study of Miller et al., 68% of the women belonged to this cluster [37].

The data on voiding frequency over 24 hours and at night showed congruence in the subjective (ICIQ-OAB) and the objective metrics (3-day bladder diary). This confirms the robustness of the chosen methodology in assessing the bladder parameters. In our collective of postmenopausal women, the specific nocturia and OAB parameters, such as the nocturnal polyuria index (42.7% before treatment, pathologic results in 35/43 women), point to disturbed atrial natriuretic factor excretion, which cooccurs with ageing, renal tubular dysfunction, and peripheral edemas. The NPi improved during treatment but still remained pathological at 39.2% (pathologic value of >33% in persons >65 years) [34]. The nocturia index (Ni) and the nocturnal bladder capacity index (NBCi) were also both pathological, which is a typical finding in women with OAB and neurogenic hyperactive bladder, although neurogenic hyperactive bladder was an exclusion criterion for the study. Although bladder function indexes were clearly pathologic, women might have adapted to a certain degree to these conditions and did not have marked sleep disorders as the baseline values from the PSQI and ESS scored moderately.

At the start of treatment, the average PSQI in the study population was 7.7, which corresponds to moderate sleep problems (0–21 scale, cut-off of >5). Nevertheless, a statistically significant improvement in sleep quality during treatment with Bryophyllum 50% chewable tablets was observed (PSQI decrease to 6.6; *p* = 0.004). Such a reduction is likely to be clinically relevant, even though most patients did not attain values characteristic of good sleepers at the end of the study (at the beginning of the study, 31 patients had a PSQI >5, and after treatment, 28 did so). The improvement was less marked than the 3-point improvement observed in a previous study with cancer patients (*n* = 20) [27]; here, the PSQI decreased from 12 to 9 during a 3-week treatment with Bryophyllum 50% chewable tablets (doses as in the present study). It is also interesting to compare the effects of Bryophyllum 50% chewable tablets on PSQI with those observed in other studies conducted with different herbal preparations but comparable populations. The effect of Bryophyllum 50% chewable tablets in the present study seemed to be less strong than the effects observed in a group of perimenopausal women with insomnia treated with Tianwang buxin granules (*n* = 13; PSQI decreased from 15 to approximately 13) [38] and in a group of postmenopausal women treated with jujube seed capsules (*n* = 53; PSQI improved from 10 to 6) [39]. This somewhat weak effect might be related to the still present nocturia condition (2.3 ± 1.3 voids per night) at the end of the study and a modest increase in nocturnal bladder capacity.

Subdomains of the PSQI questionnaire showed significant improvements in subjective sleep quality, sleep latency, and daytime dysfunction. The two first components, sleep quality and sleep latency, were improved as well in the previous study with cancer patients, in which, in addition, habitual sleep efficiency, sleep disturbance, and use of sleeping medication were positively influenced. However, daytime dysfunction was not improved in cancer patients, which might be due to cancer-induced fatigue. In an earlier prospective clinical study of the effect of Bryophyllum 50% chewable tablets on sleep quality during pregnancy, significant improvements in subjective sleep quality and a reduction in sleep disturbances were detected (*n* = 49) [26]. Taken together, the three studies indicate that the use of Bryophyllum 50% chewable tablets is effective for multiple types of sleeping problems, but depending on patients' comorbidities and the seriousness of their sleep disorders, slightly different aspects of sleep quality might be improved.

A weakness of the study is the lack of a placebo group or randomisation and the lack of a longer follow-up, either by a longer intake of *B. pinnatum* or a second follow-up assessment after drug intake to catch the long-term efficacies. A follow-up visit was carried out in the earlier OAB study six weeks after drug intake, which showed a diminution of the effect (i.e., under treatment, a −26% decrease, and 6 weeks after treatment, a −13% reduction in basal micturition frequency/24 hours) [31].

The women showed high compliance with the study drug (>90%), probably due to good tolerance of the tablets and a positive individual effect. Only two events of suspected adverse drug reactions had to be recorded (stomach pain and whole-body itching). This low number is congruent with our previous study, where the adverse events in the Bryophyllum and placebo groups were comparable [31].

## 5. Conclusion

Bryophyllum 50% chewable tablets can be regarded as a good, well-tolerated, and high-compliance alternative for the treatment of nocturia, expanding the repertoire of standard nocturia management. The data from this study encourage further clinical investigation into the use of Bryophyllum 50% chewable tablets in the treatment of different aspects of the OAB syndrome.

## Figures and Tables

**Table 1:** Patient characteristics, intake of Bryophyllum 50% chewable tablets, and discomforts possibly caused by this study medication.

	*N* or mean ± SD
Age (years)	68.5 ± 11.6
BMI	27.4 ± 6.5
Nicotine user	8/49
Current additional medications (y/n)	30/49
Parity (≥1)	33/49
Spontaneous	70
Forceps	1
C-sections	6
Average number of children	2.3 ± 1.16
Tablet intake (*n* = 48)	79.1 ± 9.8
Forgotten tablets (*n* = 48)	4.9 ± 9.8
Adverse events possibly caused by Bryophyllum 50% tablets	8

**Table 2:** Nocturia (3-day voiding diary) and sleep quality (PSQI) before and after treatment with Bryophyllum 50% tablets. ^*∗*^= statistically significant.

	Before treatment mean ± SD	After treatment mean ± SD	*p* value
Nocturia, voids/night (*n* = 43)	3.2 ± 1.4	2.3 ± 1.3	<0.001^*∗*^
PSQI score (*n* = 45)	7.7 ± 3.7	6.6 ± 3.4	0.004^*∗*^
Subjective sleep quality (*n* = 45)	1.5 ± 0.8	1.2 ± 0.7	0.008^*∗*^
Sleep latency (*n* = 45)	1.4 ± 0.9	1.2 ± 1.0	0.062
Sleep duration (*n* = 45)	0.8 ± 1.0	0.6 ± 0.9	0.031^*∗*^
Habitual sleep efficiency (*n* = 45)	1.0 ± 1.3	1.0 ± 1.2	0.890
Sleep disturbance (*n* = 45)	1.3 ± 0.5	1.1 ± 0.5	0.800
Use of sleep medication (*n* = 45)	0.5 ± 1.0	0.4 ± 1.0	0.261
Daytime dysfunction (*n* = 45)	1.2 ± 0.9	1.0 ± 0.8	0.040

**Table 3:** ICIQ score and ESS before and after treatment with Bryophyllum 50% tablets. ^*∗*^= statistically significant.

	Before treatment mean ± SD	After treatment mean ± SD	*p* value
ICIQ-OAB (*n* = 42)	8.60 ± 2.889	6.98 ± 3.072	<0.001^*∗*^

Voiding/24 hrs (*n* = 43)	1.77 ± 1.45	1.44 ± 1.20	0.046^*∗*^
0: 1–6x/24 hrs			
1: 7-8x/24 hrs			
2: 9-10/24 hrs			
3: 11-12/24 hrs			
4:>13x/24 hrs			
Voiding/night (*n* = 43)	3.23 ± 0.92	2.63 ± 1.16	0.001^*∗*^
0: 0x/night			
1: 1x/night			
2: 2x/night			
3: 3x/night			
4:> 4x/night			
Urge (*n* = 43)	2.14 ± 1.06	1.81 ± 1.18	0.085
0: Never			
1: Rarely			
2: Sometimes			
3: Mostly			
4: Always			
Incontinence (*n* = 43)	1.37 ± 0.98	1.16 ± 1.09	0.118
0: Never			
1: Rarely			
2: Sometimes			
3: Mostly			
4: Always			
ESS	6.80 ± 4.13	5.45 ± 4.27	0.015^*∗*^

**Table 4:** Further specific micturition characteristics determined from the 3-day voiding diaries before and after treatment with Bryophyllum 50% tablets.

	Before treatment mean ± SD	After treatment mean ± SD	*p* value
Volume urine/micturition (ml, *n* = 43)	179.7 ± 60.7	188.5 ± 75.0	0.070
Volume urine night/micturition (ml, *n* = 41)	206.0 ± 112.0	209.2 ± 107.8	0.753
Fluid intake/24 h (ml, *n* = 43)	1819.6 ± 671.3	1723.6 ± 764.6	0.289
Micturition/24 h (*n* = 43)	10.3 ± 3.1	8.6 ± 2.5	≤0.001^*∗*^
Urge intensity (*n* = 36)	2.1 ± 0.5	1.9 ± 0.5	0.031^*∗*^
Urge episodes (*n* = 41)	6.9 ± 4.7	6.1 ± 4.0	0.111
Incontinence episodes (*n* = 42)	1.3 ± 2.3	1.2 ± 2.6	0.753
Nocturia index (Ni, *N* = 43)	2.7 ± 0.7	2.2 ± 0.8	<0.001^*∗*^
Nocturnal polyuria index (NPi, *N* = 43)	42.7 ± 12.7	39.2 ± 15.0	0.034^*∗*^
Nocturnal bladder capacity index (NBCi, *N* = 43)	1.5 ± 0.9	1.2 ± 0.9	0.036^*∗*^

## Data Availability

Data will be available after publication via open access on the University of Zurich Open Access Repository and Archive (ZORA). https://www.zora.uzh.ch/. As soon as the manuscript will be published, it will be stored in the ZORA open repository along with the data file.
